# Hydroxypropyl Cellulose Assembled Microspheres as Structural Color Barcodes from Revolving Microfluidics

**DOI:** 10.1002/advs.202506556

**Published:** 2025-06-04

**Authors:** Qiao Wang, Chong Wang, Zhonglin Fang, Zhuohao Zhang, Ye Zhao, Teng Ma, Luoran Shang

**Affiliations:** ^1^ Shanghai Xuhui Central Hospital Zhongshan‐Xuhui Hospital and the Shanghai Key Laboratory of Medical Epigenetics The International Co‐laboratory of Medical Epigenetics and Metabolism (Ministry of Science and Technology) Institutes of Biomedical Sciences Fudan University Shanghai 200032 China; ^2^ Department of Gastroenterology Ruijin Hospital Shanghai Jiao Tong University School of Medicine Shanghai 200025 China; ^3^ Department of Thoracic Surgery Zhongshan Hospital Fudan University Shanghai 200032 China

**Keywords:** barcodes, cholesteric liquid crystals, hydroxypropyl cellulose, microfluidics, structural color

## Abstract

Optical barcodes are versatile information carriers widely applied for encryption, commercial anti‐counterfeiting, and biomedical fields. Hydroxypropyl cellulose (HPC), as a natural derivative, exhibits excellent biocompatibility and can self‐assemble into cholesteric liquid crystals (CLCs) with structure color. However, the high viscosity of HPC CLCs is a huge hurdle for material processing and thus limits their applications. In this study, a high‐speed revolving microfluidic platform is developed for emulsifying high‐viscosity methacrylate functionalized HPC (HPC‐MA) solution to form droplets. HPC‐MA molecules in the droplets can self‐assemble into CLCs by water evaporation, and the resultant CLCs droplets can be cross‐linked to form structural color barcode particles. The prepared HPC‐MA CLCs barcoded particles exhibit well‐defined and adjustable encoding information while maintaining excellent biocompatibility. Furthermore, the prepared barcode particles also demonstrate great potential in 3D cell culture and multiplex immunoassays. This work introduces an efficient way to continuously produce HPC‐MA CLCs barcode particles with finely tunable size and uniformity. Such barcode particles are promising for widespread applications in bioanalysis and biodiagnostics.

## Graphical abstract

A high‐speed revolving microfluidic platform is developed for emulsifying high‐viscosity HPC‐MA solution to form droplets, which can be self‐assembled into CLCs by water evaporation and cured as structure color particles with well‐defined and adjustable encoding information while maintaining excellent biocompatibility. The prepared barcode particles demonstrate great potential in multiplex immunoassay applications.

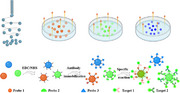

## 1 Introduction

Barcode particles are special micro/nanocarriers that encode unique information on their surface or interior. Such information marking and identification capabilities make barcode particles have wide‐ranging applications in biomedical fields such as multiplex assays, cell labeling, drug screening, etc.^[^ ^1^, ^2^, ^3^, ^4^, ^5^, ^6^ ^]^ Within the various encoding mechanisms, optical encoding is among the most convenient and accurate methods because of its facile encoding and decoding processes.^[^ ^7^, ^8^ ^]^ Although a lot of optical barcodes have been developed, most of them rely on using synthetic materials such as fluorescent dyes, upconversion nanoparticles, quantum dots, and colloidal photonic crystals as distinguishable tags, whose complex preparation process and undetermined biosafety restrict their practical applications.^[^ ^9^, ^10^, ^11^ ^]^ Therefore, the construction of barcode particles based on natural materials is still highly desirable.

**Figure 1:**
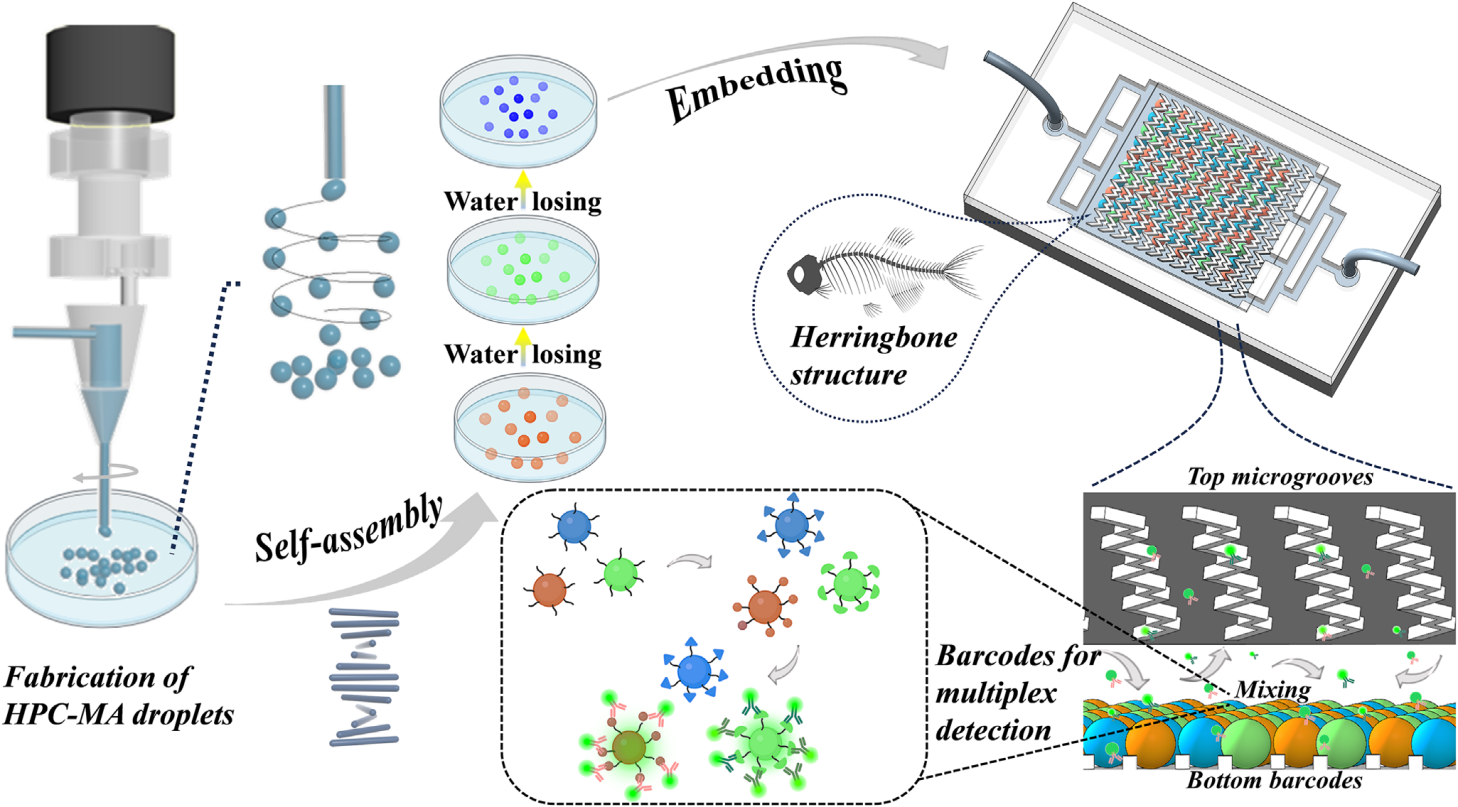
Schematic showing the preparation of HPC‐MA CLCs‐based barcode particles and their application for bioanalysis in a herringbone‐structured mixer chip. Created with BioRender.com.

In this paper, we propose novel natural cellulose‐based cholesteric liquid crystals (CLCs) structural color barcode particles for multiplex detection by a high‐speed revolving microfluidic platform, as shown in **Figure** **1**. Cellulose is a biopolymer widely abundant in nature. Hydroxypropyl cellulose (HPC) as its derivatives can be self‐assembled into CLCs in the concentration range of 50–70 wt.%, whose periodic physical structure enables its interaction with light to produce structural colors that are bright, tunable, and easy to identify.^[^ ^12^, ^13^, ^14^ ^]^ Notably, compared with other types of structural color materials such as colloidal photonic crystals, HPC has well‐established biocompatibility since it is edible and thus has great potential for biomedical applications.^[^ ^15^, ^16^, ^17^ ^]^ Meanwhile, microfluidics is a technology that excels in controlling fluids in microscale channels and can continuously produce emulsion droplets as soft templates for fabricating microparticles.^[^ ^18^, ^19^, ^20^ ^]^ Compared with other emulsification methods, microfluidic‐derived particles show finely controlled structures and exceptional monodispersity.^[^ ^21^, ^22^, ^23^ ^]^ However, processing HPC CLCs into droplets by microfluidics, and the same for other emulsification methods, is extremely challenging due to their high viscosity. The requirement for sufficient fluid shear for droplet pinch‐off may even damage the microchannels.

Herein, we established a high‐speed revolving microfluidic platform by combining a motor system and a microfluidic channel. This type of microfluidic setup has the potential to generate droplets from liquids with special physical properties. Here, with the circular translational motion of the needle, a stream of methacrylate functionalized HPC (HPC‐MA) solution in a certain concentration range was broken up into uniform droplets under the drag force and interfacial tension. With this, the morphology and generation rate of HPC‐MA droplets can be well manipulated by the revolving speed and flow rate. The as‐prepared HPC‐MA droplets were converted into CLCs with structure colors by HPC self‐assembly during water loss and further cross‐linked to form barcode particles under UV irradiation. The barcode particles have bright, well‐defined structured color information as well as good biocompatibility. Additionally, acrylic acid (AAc) was added to the HPC‐MA prepolymer to make the prepared barcode particles contain functional carboxyl groups on the surface, which facilitates antigen or antibody attachment. Finally, we embedded the prepared barcode particles in a herringbone mixer chip and demonstrated their efficiency for multiplexed immunoassay. This work introduces an efficient strategy for the preparation of HPC CLCs barcodes and represents a leap in pushing HPC photonic materials forward for bio‐diagnostics applications.

## 2 Results and Discussion

In a typical experiment, the high‐speed revolving microfluidic platform was constructed using a motor sleeve, revolving microchannel, motor bracket, built‐in bracket, a direct current (DC) supply, and a motor, as shown in Figure S1 (Supporting Information). The inlet and outlet of the revolving microchannel are equipped with stainless steel capillaries and flat‐tip needles for delivering the dispersed phase. The built‐in bracket is employed for holding the continuous phase bath. The motor sleeve and revolving microchannel constitute a motion body by a ball bearing, which can avoid hose entanglement. The DC motor was used to drive the revolving microchannel to perform a high‐speed revolving motion. Using a syringe pump, the dispersed phase was propelled into the outlet needle tip of the revolving microchannel, which, when operated at a constant revolving motion, facilitates the formation of uniformly sized droplets in the collection phase.

**Figure 2:**
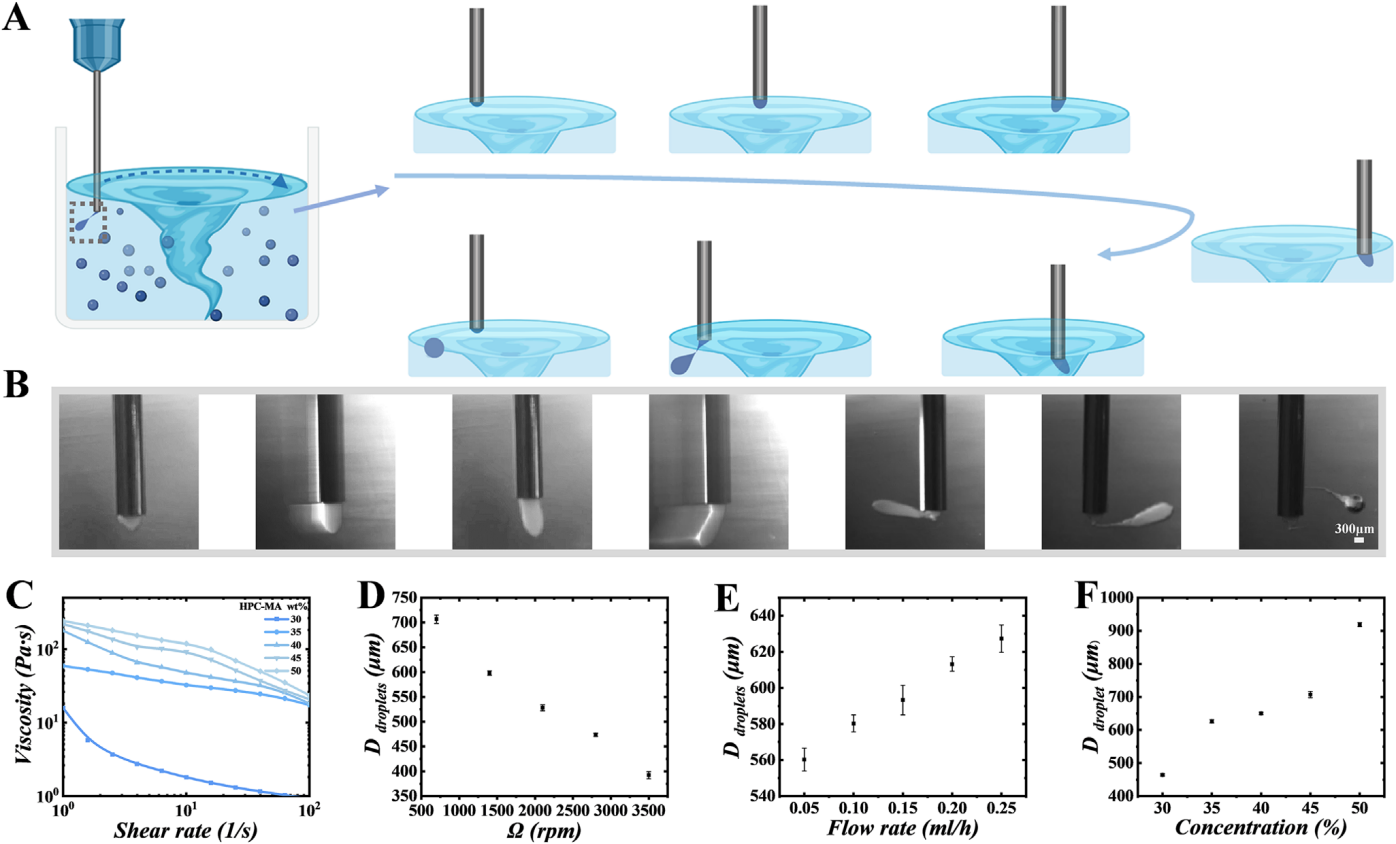
Preparation of HPC‐MA droplets. A) Schematic and B) real‐time images showing the generation of HPC‐MA droplets via the revolving motion of the channel. C) Shear viscosity profiles of the HPC‐MA solutions at 30–50 wt.% concentration. D) Effect of revolving speed on the size of HPC‐MA droplets under a dispersion phase with a flow rate of 0.15 mL h^−1^ and 40 wt.% concentration of the HPC‐MA solution. E) Impact of dispersed phase solution flow rate on droplet size at the revolving speed of 1500 rpm and 40 wt.% concentration of the HPC‐MA solution. F) Impact of the concentration of dispersed phase solution on the droplet size at the revolving speed of 1500 rpm and a dispersion phase flow rate of 0.3 mL h^−1^. Created with BioRender.com.

A high‐concentration HPC‐MA aqueous solution was used as the dispersed phase, which was doped with carbon nanotubes (CNTs) to enhance the color saturation. Paraffin oil containing 4 wt.% sorbitan monooleate (Span 80) was used in the collection phase. The viscous paraffin oil was expected to provide the necessary shear for droplet pinch‐off. Span 80 was added as a surfactant to prevent HPC‐MA droplet coalescence. The production process of HPC‐MA droplets is depicted in **Figure** **2**A,B. When the dispersed phase of HPC‐MA flows from the syringe to the outlet needle tip of the revolving body, a tethered droplet is formed, which elongates and gradually grows in volume while being subjected to the drag force. Subsequently, the tethered droplets deform until they break up to form uniformly dispersed droplets when the drag force acting on the dispersed phase exceeds the surface tension.

High‐concentration HPC‐MA solutions exhibit non‐Newtonian fluid properties, as indicated by the decrease in viscosity with shear rate (Figure 2C). We next investigated whether the concentration of HPC‐MA affects the morphology of the generated droplets. We prepared HPC‐MA solutions in the concentration range of 35–60 wt.% and observed the droplet shape with microscopy. We found that the droplets generated from 35–50, 55, and 60 wt.% HPC‐MA were spherical, slightly ellipsoidal, and elongated teardrop‐shaped (Figure S2, Supporting Information). Such deformation may be attributed to the viscoelasticity of the HPC‐MA dispersed phase.^[^ ^24^ ^]^ Additionally, we studied the dimensions of the droplets as influenced by the parameters of the microfluidic platform. We anticipated that the size of the droplets should depend on the revolving speed of the revolving microchannel, the dispersed‐phase flow rate of the solution, and the concentration of the HPC‐MA solution. Therefore, size analysis was performed on the droplets generated by modulating these variables in the platform using a dispersed phase with a concentration in the range that leads to the production of spherical droplets. Under certain conditions, the droplet size can be observed to decrease with the increase of the revolving speed and increase with the flow rate and concentration of the dispersed phase (Figure 2D–F).

**Figure 3:**
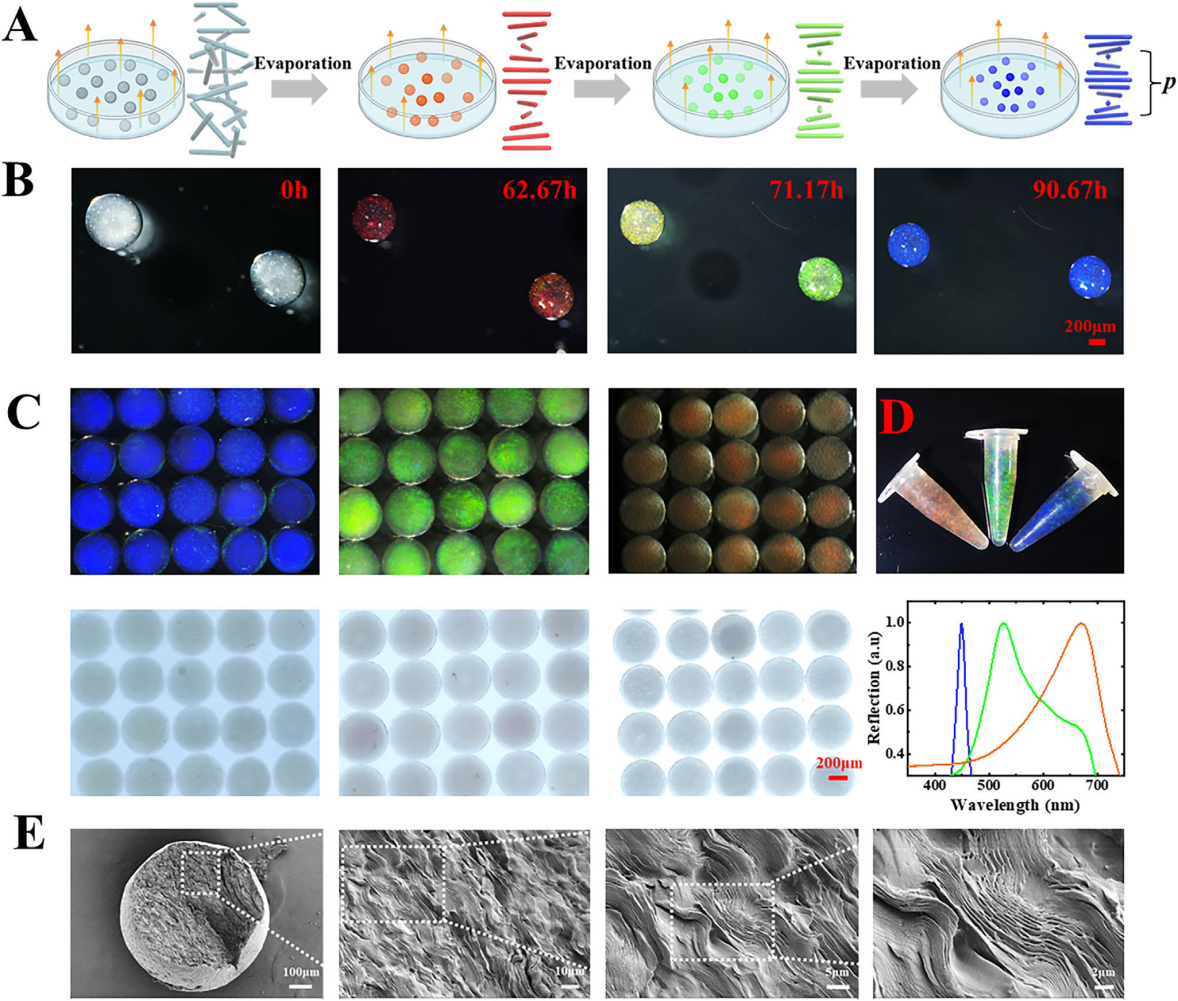
Characterization of HPC‐MA CLCs structural color particles. A) Schematic diagram and B) real‐time images showing HPC‐MA self‐assembled into CLCs in the droplets upon water loss. C) Representative microscopic images of R, G, and B HPC‐MA CLCs particles under reflected (top) and transmitted (bottom) light. D) Image of a batch of R, G, and B HPC‐MA CLCs particles collected in EP tubes (top) and their corresponding reflective spectra (bottom). E) SEM images of R HPC‐MA CLCs particles in different magnifications. Created with BioRender.com.

The HPC‐MA self‐assembly process in the droplet was driven by continuous water loss and was monitored using an optical microscope. We found that the volume of 50 wt.% HPC‐MA droplets gradually decreased under room temperature and appeared red (R) colors after almost 60 h. Meanwhile, its red color gradually transitions to green (G) and blue (B) as the resting time increases, as shown in **Figure** **3**A,B. This is because HPC is a long‐chain molecule with hydroxyl groups and can self‐assemble to form CLCs with a periodic arrangement in the higher concentration range.^[^ ^25^, ^26^, ^27^ ^]^ Under room temperature, the HPC‐MA droplets lost water gradually, resulting in the size of the droplets decreasing and the molecular concentration of HPC‐MA within the droplets increasing, facilitating them to self‐assemble into CLCs. The self‐assembled CLCs are typically 1D photonic crystals having a photonic bandgap, resulting in selectively reflecting the wavelengths within the bandgap.^[^ ^28^, ^29^, ^30^ ^]^ Therefore, when the characteristic pitch *p* of the HPC‐MA CLC matches the wavelength of visible light, the HPC‐MA CLC droplets reflect bright structural colors. In addition, the continuous loss of water from HPC‐MA CLCs gradually decreases the *p*‐value, resulting in a blue shift of the structural colors. It's worth noting that the heating conditions can accelerate the assembly of HPC‐MA molecules in the droplets, but the droplets became pale during the heating process, and the droplets needed to be cooled down at room temperature for a period of time for their structural color to be presented, as shown in Figures S3, S4 (Supporting Information). In addition, the prepared HPC‐MA CLCs droplets possess temperature sensing and mechanical sensing properties, as shown in Figure S5 (Supporting Information).

The self‐assembled HPC‐MA CLCs droplets were cross‐linked and solidified into structure color‐barcoded particles under UV light. In addition, the irradiation time of UV did not affect the color retention of HPC‐MA CLCs particles. As shown in Figure S6 (Supporting Information, there was no significant change in the structure color information before and after UV curing. Barcode particles with six types of colors are presented in Figure S7 (Supporting Information), and representative R, G, and B HPC‐MA CLCs barcode particles were further characterized. As shown in Figure 3C, the as‐prepared HPC‐MA CLC microspheres exhibit bright and identifiable structural colors. The reflective spectra of the prepared barcode particles were measured using a spectrometer, and the reflection wavelengths of R, G, and B structural color barcode particles were 670, 527 and 448 nm, respectively, corresponding to their color appearance. Moreover, we found that the structure color and morphology of HPC‐MA CLCs structure color barcoded microparticles did not change significantly after 7 days of immersion in water, PBS, serum, culture medium, simulated colonic fluid, and simulated gastric fluid, indicating that HPC‐MA CLCs structure color barcoded microspheres have excellent long‐term stability (Figure S8, Supporting Information). The scanning electron microscope (SEM) results in Figure 3E and Figure S9 (Supporting Information) show a periodic microstructure inside the barcode particles, validating the origin of the structural colors.

It is worth mentioning that high‐concentration HPC droplets are very difficult to form in common co‐flow capillary microfluidic devices due to their high viscosity. As shown in Figure S10A (Supporting Information), HPC‐MA solutions at a concentration above 25 wt.% tend to form a continuous jet or exhibit non‐uniform breakup in the co‐flow microchannel, making it difficult for the preparation of monodispersed HPC‐MA droplets. Although HPC‐MA solutions at a much lower concentration, e.g., below 10 wt.% can pinch‐off into uniform droplets in the co‐flow microfluidic channel, such low concentration is not conducive to the subsequent self‐assembly of HPC molecules, and the final produced CLCs particles exhibit an uneven distribution of structural color and morphological deformation (Figure S10B, Supporting Information). In this sense, the revolving microfluidic platform provides a more robust way of fabricating HPC structural color barcodes.

**Figure 4:**
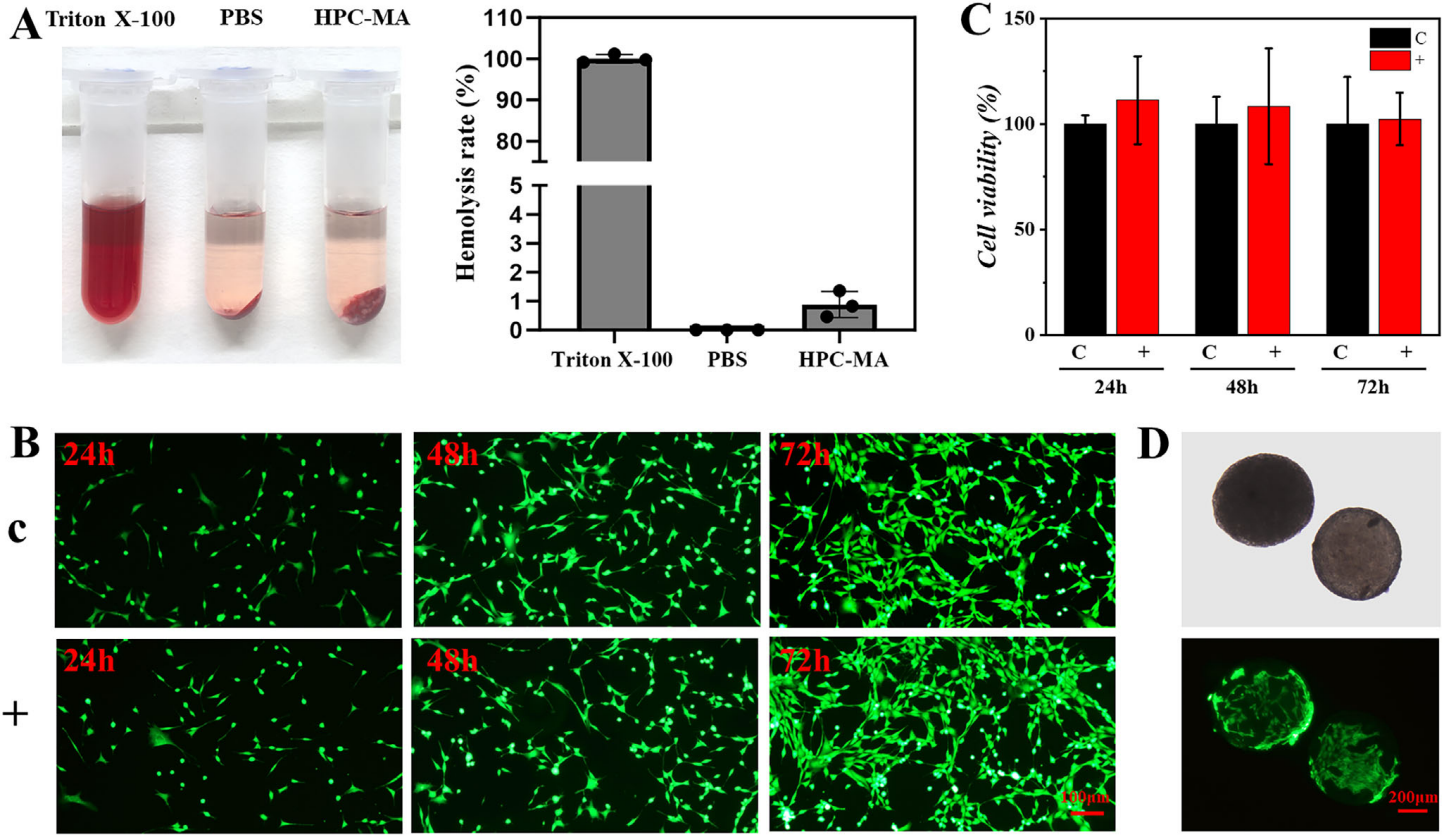
Biocompatibility tests. A) Digital photographs (left) and corresponding hemolytic ratios plot (right) of erythrocytes treated with Triton X‐100, PBS, and HPC‐MA CLCs particles. B) Fluorescence images of NIH 3T3 cells stained with Calcein‐AM after 24, 48, and 72 h of incubation in medium and medium with leachate of HPC‐MA‐GelMA CLCs particles. C) Plot of the viability of NIH 3T3 cells after 24, 48, and 72 h of incubation in medium and medium with leachate of HPC‐MA/GelMA particles. The C group was NIH 3T3 cell culture in medium (as the control group), and the + group was NIH 3T3 cell culture in medium with the leachate of HPC‐MA/GelMA particles (as the experimental group). D) Bright‐field microscopic (top panel, under transmitted light) and fluorescence images (bottom panel) of HPC‐MA/GelMA microspheres co‐cultured with NIH 3T3 cells for 2 days. (Scale bars: 100 µm in C; 200 µm in D.).

To evaluate the biocompatibility of the HPC‐MA CLCs barcode particles, a hemolysis test was performed. The hemolysis rate was determined as 0.9%, reflecting good hemocompatibility, as shown in **Figure** **4**A. In addition, HPC‐MA CLCs barcode particles were doped with gelatin methacryloyl (GelMA) by using GelMA‐doped dispersed phase HPC‐MA solution when preparing. The leachate of the resultant HPC‐MA/GelMA barcode particles was co‐cultivated with NIH 3T3 cells for 72 h. The cells cultured in the medium were set as the control group, and those cultured in the medium with the leachate of the HPC‐MA/GelMA barcode particles were the experimental group. We viewed the state and morphology of NIH 3T3 cells at 24, 48, and 72 h in both groups by staining with Calcein‐AM and observing under fluorescence microscopy, which showed almost identical growth status (Figure 4B). Cell counting kit‐8 (CCK‐8) assays were also conducted to examine the cell viability between control groups of medium and experimental groups of leachates, which indicated no statistically significant difference between them (Figure 4C). These results confirmed the excellent biocompatibility of the barcode particles. The spherical structure and controllable size of the barcode particles are suitable for 3D cell culture. Furthermore, the doping of GelMA is favorable for cell adhesion.^[^ ^31^, ^32^, ^33^ ^]^ To validate this, the HPC‐MA/GelMA barcode particles were co‐incubated with NIH 3T3 cells for 2 days and subsequently subjected to live cell staining. The results of fluorescent staining showed that NIH 3T3 cells can proliferate and grow normally on the HPC‐MA/GelMA barcode particles (Figure 4D), suggesting that the prepared barcode particles have potential in the use of cell assays and drug screening.

**Figure 5:**
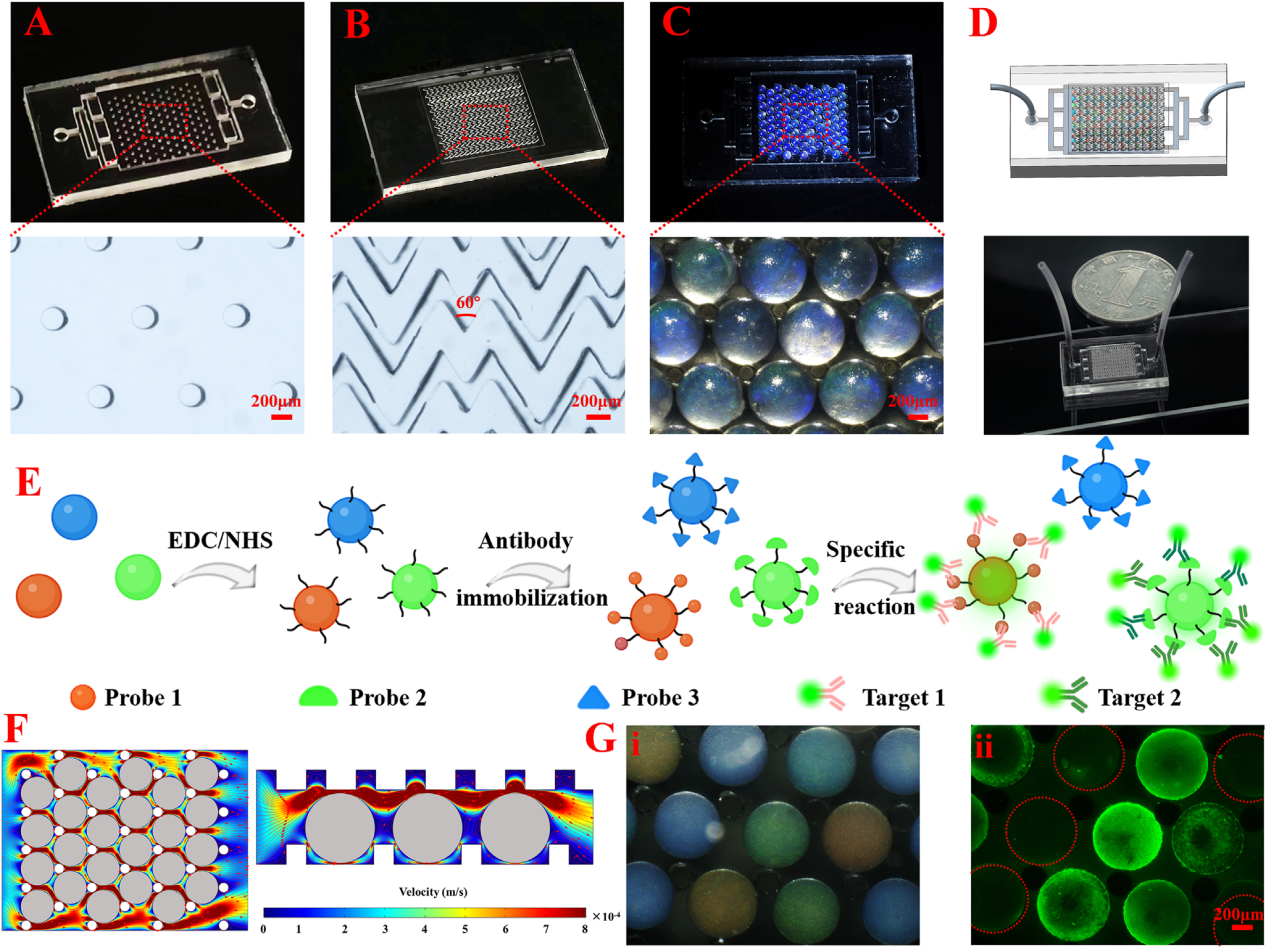
Multiplexed bioanalysis of HPC‐MA CLCs barcode particles on herringbone microfluidic chips. A) Photograph (top) and optical microscopy image (bottom) of the bottom layer with a periodically arranged micropillar array structure. B) Photograph (top) and optical microscopy image (bottom) of the top layer with a periodic herringbone‐like array structure. C) Photograph (top) and optical microscopy images (bottom) of B HPC‐MA CLCs barcode particles embedded in between the microcolumn arrays. D) Schematic diagram (top) and the photograph (bottom) of the integrated microfluidic chip. E) Schematic illustration of the HPC‐MA CLCs barcode particles for multiplexed immunoassays. F) Vertical (left) and lateral views (right) of flows in the PDMS integrated chip. G) Bright‐field microscopic i) and fluorescence images ii) of HPC‐MA CLCs barcode particles after specific reaction to target molecules on‐chip. (Scale bars: 200 µm in A, B, C, G). Created with BioRender.com.

To validate the application of the HPC‐MA CLCs barcode particles in multiplex detection, the R, G, and B HPC‐MA CLCs barcode particles were embedded in a polydimethylsiloxane (PDMS) microfluidic chip for immunoassay experiments. The microfluidic chips were constructed via UV bonding of a bottom layer containing micropillar arrays and a top layer containing herringbone‐shaped microchannels, as shown in **Figure** **5**A–D. The top herringbone layer consisted of microgrooves at an angle of 60° and matched to the dimensions of the bottom layer where the HPC‐MA CLCs barcode particles were embedded. The micropillar arrays in the bottom layer accommodated HPC‐MA CLCs barcode particles at equal intervals to ensure that the particles could be immobilized. The herringbone grooves of the top layer promote fluid turbulence. Therefore, the periodic microcolumn and angled groove structure resulted in fluid mixing inside the microfluidic chip, enhancing contact between the particles and target biomolecules, as validated by the fluid streamlines obtained by numerical simulation (Figure 5F).

We tested the ability of HPC‐MA barcode particles embedded in the chip to detect target molecules in sample solutions. AAc was added to the dispersed phase of HPC‐MA to form particles with carboxyl groups on the surface, which were activated by N‐(3‐dimethylaminopropyl)‐N′‐ethylcarbodiimide hydrochloride (EDC)/ N‐hydroxysuccinimide (NHS) and bound to antigens/antibodies via the formation of amide bonds for subsequent multiplex analyses.^[^ ^34^, ^35^, ^36^ ^]^ Next, the R, G, and B HPC‐MA barcode particles were collected and immobilized using probe 1 of human IgG, probe 2 of mouse IgG, and probe 3 of rabbit IgG. Subsequently, the immobilized particles were embedded in the microfluidic chip that was injected with target solutions containing FITC‐labeled goat anti‐human IgG secondary antibody (target 1) and FITC‐labeled goat anti‐mouse IgG secondary antibody (target 2). The unique channel design of the microfluidic chip facilitated the efficient binding of the particle surface probes to the target molecules in the sample solution. Note that only the R and G HPC‐MA barcode particles showed fluorescence under the microscopy, while the B barcode particles did not show fluorescence (Figure 5G). These results demonstrated the multiplex detection capability of the prepared HPC‐MA CLCs barcode particles.

## 3 Conclusion

In summary, we developed a revolving microfluidic platform for the generation of HPC‐MA droplets, which were then converted to CLC droplets by self‐assembly of HPC‐MA molecules and cross‐linked into structural color barcode particles. The barcode particles were then employed for multiplexed assays efficiently with the aid of a herringbone micromixer chip. The revolving microfluidic platform overcomes the limitations of traditional microfluidic chip design in terms of successfully emulsifying a high‐viscosity HPC‐MA solution, allowing for continuous generation of high‐viscosity HPC‐MA droplets with precisely controlled size, shape, and monodispersity. The prepared droplets can be converted into CLCs droplets upon water loss and HPC‐MA molecular self‐assembly, and then UV‐cured to form solid particles. The resulting HPC‐MA CLCs particles have bright structural colors, periodic internal structures, and good biocompatibility. Meanwhile, doping functional ingredients make the HPC‐MA CLCs particles have great potential applications in various fields. As an example, the incorporation of GelMA evidenced the 3D cell culture ability of the HPC‐MA CLCs barcode particles. Finally, we embedded functional HPC‐MA CLCs barcode particles containing carboxyl groups into a microfluidic chip and validated their capability in multiplexed detection. Building on these merits, we believe that the revolving microfluidic platform is anticipated to be an excellent platform for generating high‐viscosity HPC‐MA droplets, and the derived CLCs structural color barcode particles show great potential for widespread biological applications.

## 4 Experimental Section

### Materials

HPC was obtained from Nippon Soda Co., Ltd. EDC, NHS, and MA was purchased from Aladdin. CNTs (dispersed solution) were bought from XF NANO Materials Tech Co., Ltd. NaOH was purchased from Sinopharm Group Co., Ltd. PDMS was purchased from Dow Corning Corporation, USA. The 2‐Morpholinoethanesulfonic acid buffer was purchased from Macklin. Paraffin oil was purchased from Sangon Biotech Co., Ltd. AAc and Span80 were obtained from Sigma‐Aldrich. Rabbit IgG, human IgG, mouse IgG, FITC‐labeled goat anti‐mouse IgG secondary antibody, and FITC‐labeled goat anti‐human IgG secondary antibody were purchased from Yeasen Co., Shanghai, China. The 1X Phosphate‐buffered saline (PBS) solution was obtained from SolarBio Science & Technology Co., Ltd. 2% rabbit red blood cell was obtained from SenBeiJia Biological Technology Co., Ltd. Bovine serum albumin (BSA) powder was purchased from Bioss Biotechnology Co. Ltd. Triton X‐100 solution was derived from Beyotime Biotechnology Co., Ltd. Deionized water was procured using a Milli‐Q Plus 185 ultrapure water system.

### Fabrication of a High‐Speed Revolving Microfluidic Platform

The microfluidic platform was constructed using a revolving microchannel, motor bracket, motor sleeve, built‐in bracket, DC, and motor. The upper connector of the motor sleeve was embedded with a DC‐driven motor, and the other connector of the motor sleeve was connected to the revolving microchannel using a ball bearing. The microchannel has an inlet and outlet that were embedded with stainless‐steel capillary and flat‐tip needles, respectively. The construction of the microfluidic system was completed by connecting the inlet stainless‐steel capillary of the revolving microchannel to the injection phase via a polyethylene tube. Then, the motor sleeve and revolving microchannel constructed a motion body, and the revolving microchannel performed a high‐speed revolving motion and generated droplets under the drive of the DC motor.

### Synthesis of HPC‐MA

HPC‐MA was synthesized according to the protocol described in the literature.^[^ ^37^ ^]^ First, 10 g dried HPC powder was dissolved into 500 mL purified water, followed by the addition of 40 ml MA. Subsequently, 59 mL NaOH solution (0.2 g mL^−1^) was added drop by drop into the prepared solution while continuously stirring under ambient temperature (≈25 °C) overnight. The final mixture was dialyzed (water was changed twice daily) for five consecutive days, after which the mixture was freeze‐dried to obtain the HPC‐MA polymer.

### Generation of HPC‐MA Droplets

The dispersed phase consisted of 30–50 wt.% HPC‐MA with 0.05 wt.% CNTs and the collection phase consisted of paraffin oil with 4 wt.% Span80 surfactant. The dispersed phase solution of HPC‐MA was loaded into the inlet stainless‐steel capillary of the revolving microchannel using a syringe pump. The output needle was revolving, driven by a motor, resulting in the breakup of the dispersed phase solution into HPC‐MA droplets. The as‐prepared droplets were placed at room temperature for 2–5 days or heated at 60 °C for 3–8 h to allow for self‐assembly of HPC‐MA molecules into CLCs.

### Preparation and Characterization of HPC‐MA CLCs Barcode Particles

The aforementioned HPC‐MA CLC droplets were cured to form HPC‐MA CLCs barcode particles using UV irradiation for 10 min. The obtained UV‐cured particles were cleaned using n‐hexane. Particles exhibiting the same structural colors were collected in Eppendorf (EP) tubes and characterized using a high‐sensitivity spectrometer. The prepared barcode particles were immersed in ethanol for 4 h and then the cracked or ruptured particles were picked up, dried in an oven, placed on a conductive adhesive, and sprayed with a gold film. Finally, the particles were observed under an SEM.

### Biocompatibility Tests of HPC‐MA Barcode Particles

The hemolytic properties of the HPC‐MA CLCs barcode particles were studied. First, the female rabbit blood was centrifuged at 1000 rpm for 10 min. After centrifugation, the obtained pure erythrocyte solution at the bottom was collected and divided into three groups. In each group, appropriate amounts of Triton X‐100 solution, PBS, and HPC‐MA CLCs particles were added and then incubated at 37 °C for 2 h. Afterward, the mixtures were centrifuged at 1000 rpm for 15 min, then the obtained supernatants were examined for absorption at 545 nm, and then the hemolysis rate was measured. Next, a solution consisting of 35 wt.% HPC‐MA and 5 wt.% GelMA was used as the dispersion solution to obtain CLCs microspheres using the afore‐described method. The prepared HPC‐MA/GelMA CLCs barcode particles were washed multiple times using PBS and ethanol, air‐dried under UV light, and then immersed in the medium at a volume ratio of 1:10 for 12 h. NIH 3T3 cells were incubated in a medium with filtered leachate and normal medium at an initial density of 5 × 10^3^ cells per group and cultured for 3 days at 37 °C. Fluorescence staining was recorded after the cells were cultured for 24, 48, and 72 h. Additionally, an experimental group and a control group with an initial density of 10^4^ cells/ml were assessed using CCK8 assay kits after the cells had been cultured for 24, 48, and 72 h. The experimental group was cells cultured in the medium containing filtered leachate of the HPC‐MA/GelMA CLCs barcode particles, and the control group was cells cultured in the medium.

### 4.1 Cell Culture

The HPC‐MA/GelMA microspheres were co‐cultured with NIH 3T3 cells at an initial density of 10^5^ cells per well and incubated for 2 days at 37 °C. The prepared culture was stained using the Calcein‐AM Kit and observed under a fluorescence microscope.

### Fabrication of Microfluidic Mixer Chip

The top layer and bottom layer of the microfluidic mixer chip were fabricated via PDMS replication of the respective mold. The PDMS mixture was produced by mixing PDMS and curing agent in the ratio of 9:1 by volume, and then the obtained PDMS mixture was injected into the mold, which was heated at 70 °C for 1 h. The cured PDMS mixture was peeled off from the mold. Finally, a UV‐curable adhesive was applied to the bottom layer to which the top layer was attached. The adhesive was allowed to cure under UV light, after which the top and bottom layers were bonded to form the integrated chip.

### Multiplex Immunoassay Experiment

The dispersed phase containing 30–50 wt.% HPC‐MA with 0.05 wt.% CNTs was mixed with 3–5 wt.% AAc. Subsequently, the prepared R, G, and B barcode particles were separately added to three EP tubes and then washed three times with PBS. Then, a mixture containing 1 mL of PBS, 40 mg of EDC, and 60 mg of NHS was injected into the EP tube and placed at ambient temperature for 45 min. After washing with PBS three times, 1 ml human IgG (0.5 mg mL^−1^), 1 mL mouse IgG (0.5 mg mL^−1^), and 1 ml rabbit IgG (0.5 mg mL^−1^) were added to the EP tubes containing the R, G, and B barcode particles respectively, and reacted at 4 °C overnight. Next, the above barcode particles were cleaned with PBS several times and each EP tube was incubated with 5% BSA solution for 2 h. Subsequently, all the barcode particles were rinsed again using PBS and randomly embedded in between the microcolumn arrays in the bottom layer of the microfluidic mixer chip. After UV‐initiated bonding of the chip, a mixture of FITC‐labeled goat anti‐mouse IgG secondary antibody (1 mg mL^−1^) and FITC‐labeled goat anti‐human IgG secondary antibody (1 mg mL^−1^) was injected into the chip and reacted for 3 h at ambient temperature in the dark. Finally, PBS was injected to rinse, and the chip was observed under the fluorescent stereomicroscope.

### Characterization

SEM (Sigma 3000, ZEISS) was used to examine the microstructures of the HPC‐MA CLC barcoded particles. The optical images of the HPC‐MA CLC barcoded particles were captured using a stereoscopic microscope (Jiangnan, NSZ608T) fitted with a CCD camera (Sony, E3ISPM). The fluorescent images of the HPC‐MA CLC barcoded particles were recorded using a fluorescence stereomicroscope (SNZ818, Nanjing) with a CCD camera (Sony, E3ISPM). The reflection spectra were measured using a high‐sensitivity spectrometer (Ocean Optics, MAYA2000PRO‐DEEP‐UV).

## Conflict of Interest

The authors declare no conflict of interest.

## Supporting information



Supporting Information

## Data Availability

The data that support the findings of this study are available from the corresponding author upon reasonable request.
