# Supplementary material for: Hydroxypropyl Cellulose Assembled Microspheres as Structural Color Barcodes from Revolving Microfluidics
Source: Adv Sci (Weinh). 2025 Jun 4;12(32):e06556. doi: 10.1002/advs.202506556 (PMC12407317; doi:10.1002/advs.202506556)
Supplement: Supplementary file 1 — Supporting Information [file ADVS-12-e06556-s001.docx]

**Supporting Information**

**Hydroxypropyl Cellulose Assembled Microspheres as Structural Color Barcodes from Revolving Microfluidics**

Qiao Wang^a^, Chong Wang^a^, Zhonglin Fang^a^, Zhuohao Zhang^a^, Ye Zhao ^b,^*,

Teng Ma ^c,^*, Luoran Shang^a,^*

^a^ Shanghai Xuhui Central Hospital, Zhongshan-Xuhui Hospital, and the Shanghai Key Laboratory of Medical Epigenetics, the International Co-laboratory of Medical Epigenetics and Metabolism (Ministry of Science and Technology), Institutes of Biomedical Sciences, Fudan University, Shanghai, China.

^b^ Department of Gastroenterology, Ruijin Hospital, Shanghai Jiao Tong University School of Medicine, Shanghai, 200025, China

^c^ Department of Thoracic Surgery, Zhongshan Hospital, Fudan University, Shanghai, 200032, China

* Corresponding author

Email: zy12889@rjh.com.cn; [ma.teng@zs-hospital.sh.cn](mailto:ma.teng@zs-hospital.sh.cn); [luoranshang@fudan.edu.cn](mailto:luoranshang@fudan.edu.cn)


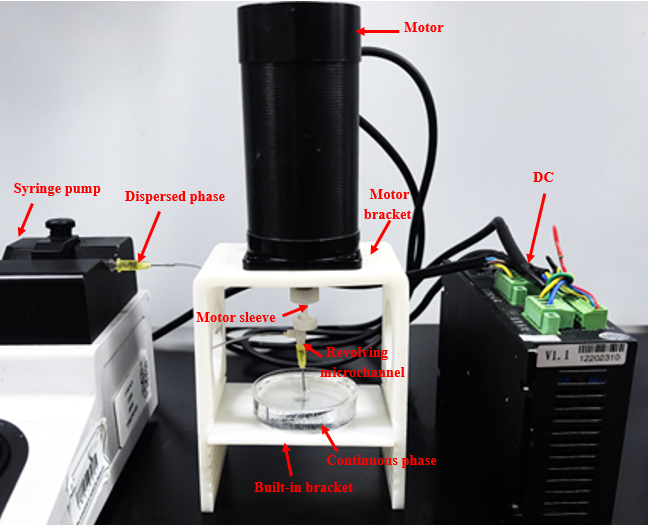


**Figure S1**. Photograph of the high-speed revolving microfluidic platform for HPC droplet formation.


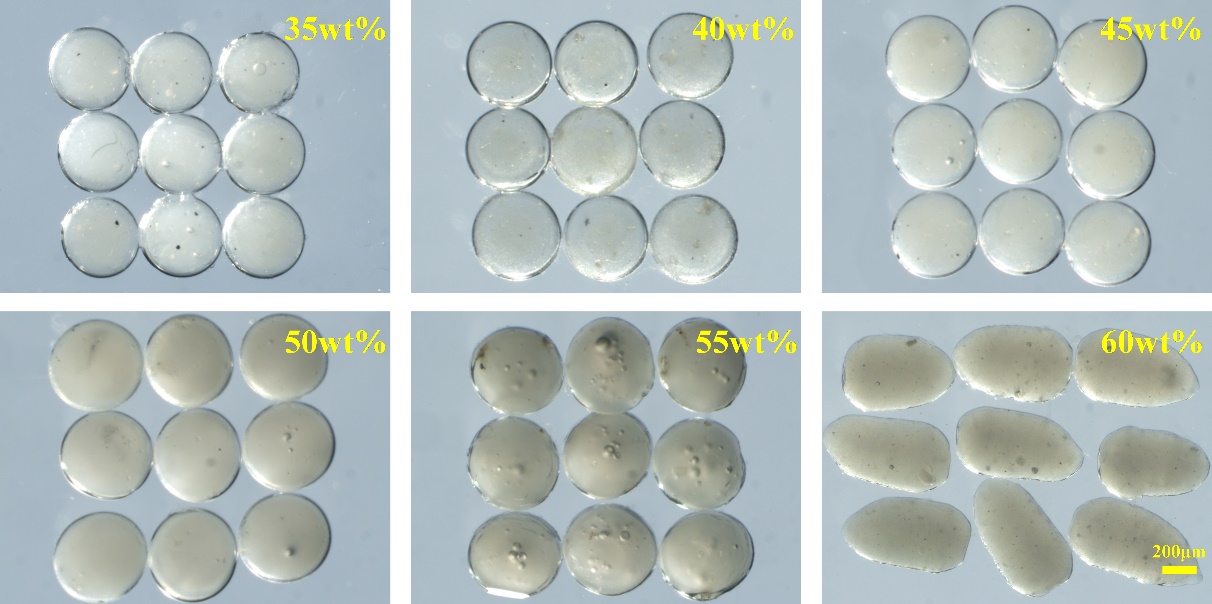


**Figure S2**. Microscopic images of droplets prepared from 35-60 wt% HPC-MA solutions at a revolving speed of 1500 rpm and a flow rate of 0.1 mL/h. (Scale bar: 200 µm).


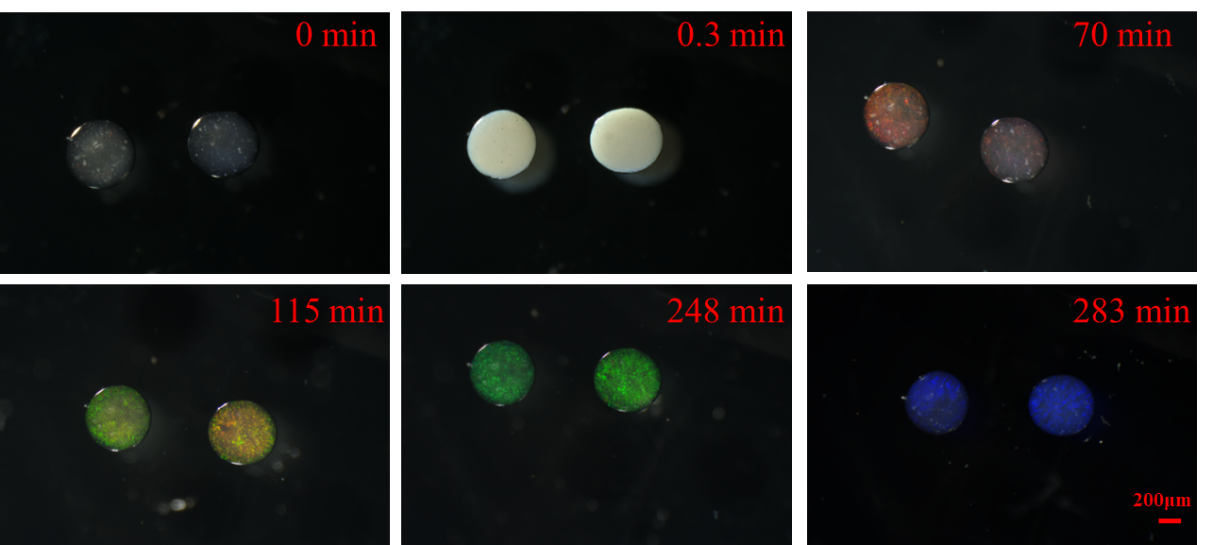


**Figure S3**. The real-time images showing HPC-MA self-assembled into CLCs in the droplets after heated at 60℃.


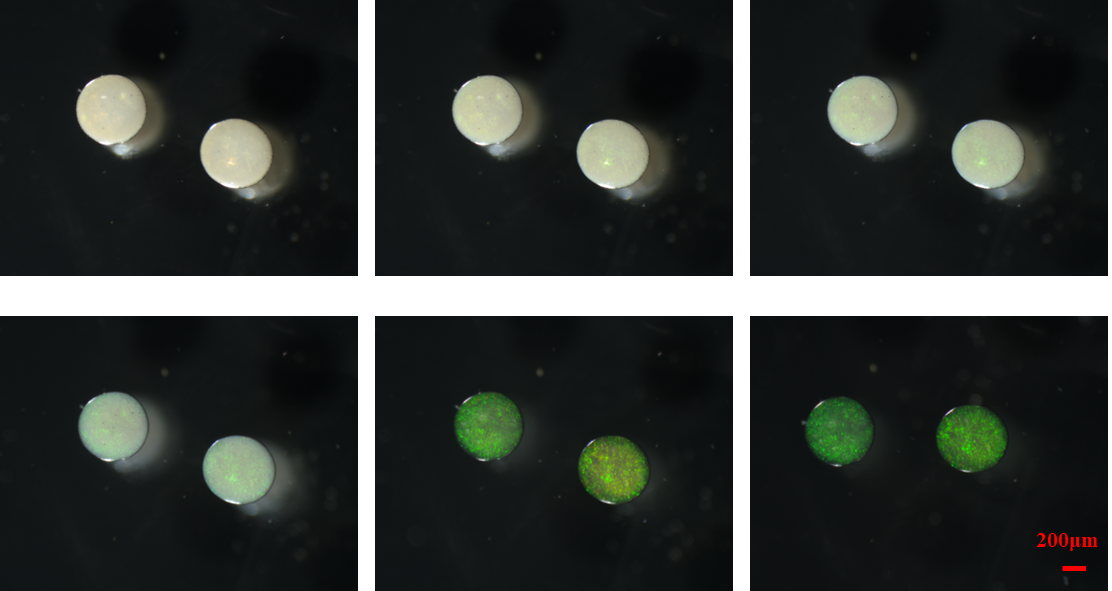


**Figure S4**. Microscopic images of HPC CLCs droplets cooling from 60℃ to room temperature


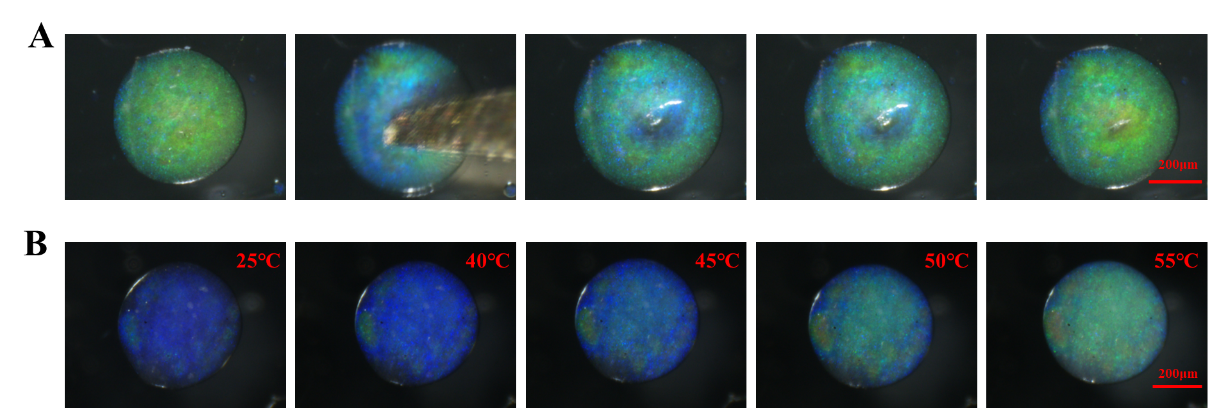


**Figure S5**. Sensing capability of the HPC-MA CLCs droplets. A) Microscopic real-time images of a G HPC-MA droplet under being pressed. B) Microscopic real-time images of a B HPC-MA droplet under heating.


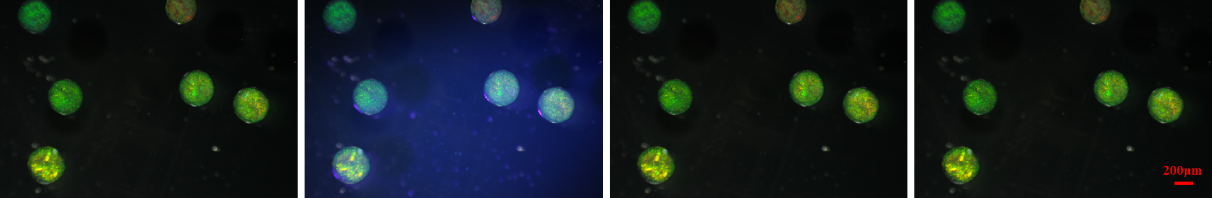


**Figure S6**. Microscopic images of HPC-MA CLCs droplets before UV irradiation, during UV irradiation, 5 mins after UV irradiation, and 10 mins after irradiation (from left to right).


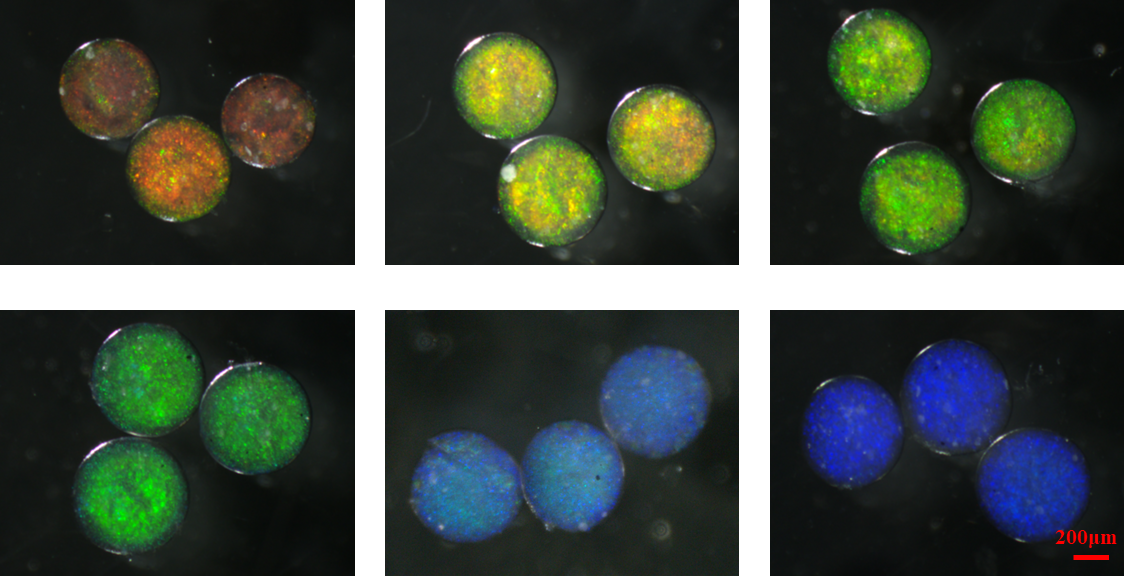


**Figure S7**. Microscopic images of red, yellow, light green, dark green, blue, and blue-violet HPC-MA CLCs barcoded particles.


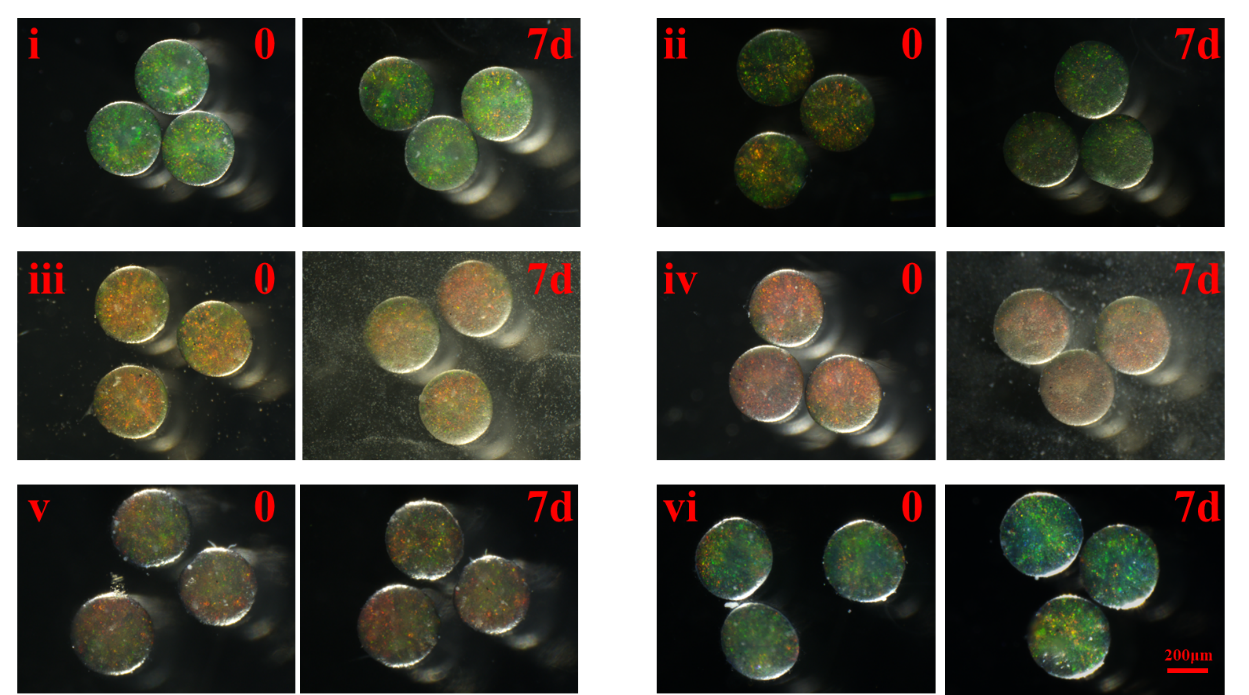


**Figure S8**. Microscope images of HPC-MA CLCs microparticles at initial status (left) and after 7d (right) immersion in water (i), PBS (ii), serum (iii), culture medium (iv), simulated colonic fluid (v), and simulated gastric fluid (vi).


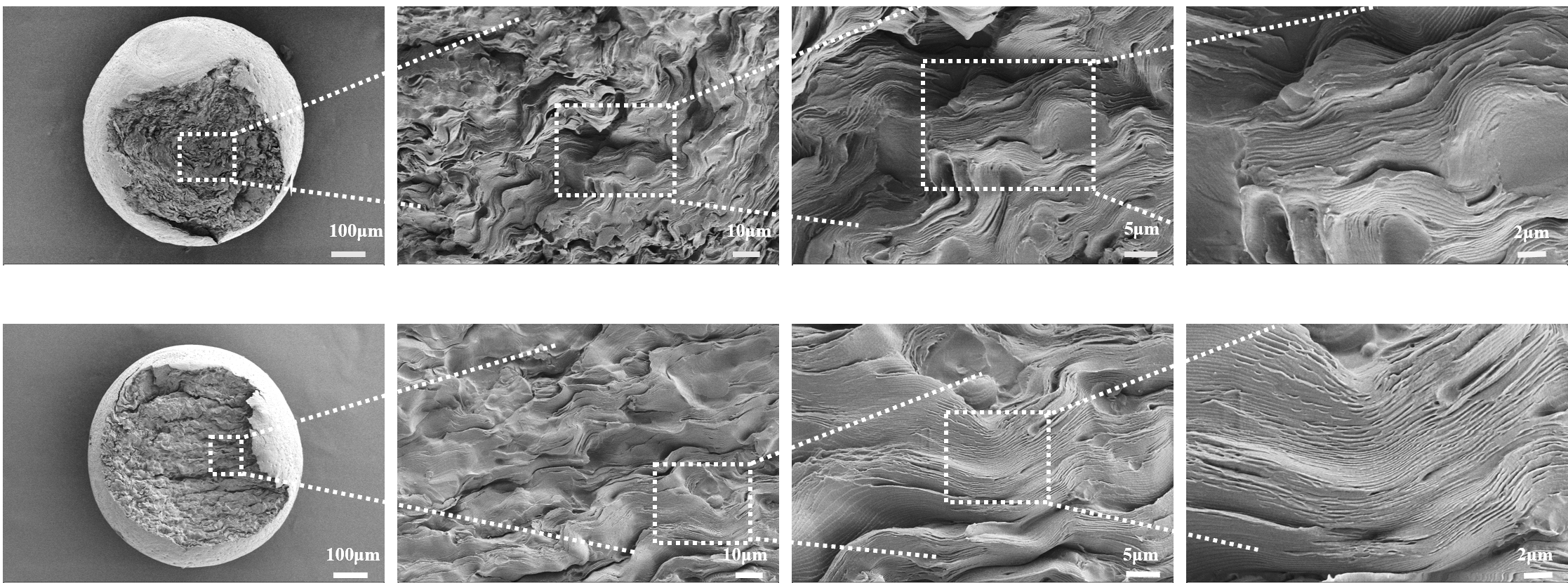


**Figure S9**. SEM images of a green (top) and blue (bottom) HPC-MA CLCs microsphere in different magnifications.


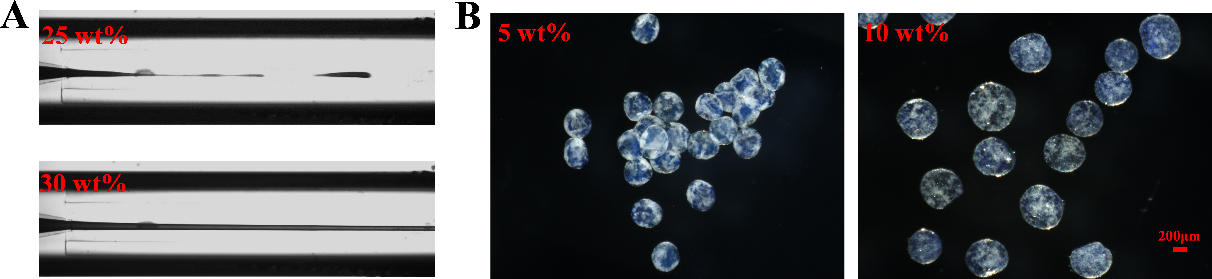


**Figure S10**. Preparation of low concentration HPC-MA droplets in the conventional co-flow capillary microfluidic device. A) Real-time images of droplets preparation in a co-flow capillary microfluidic channel using 25 wt% and 30 wt% HPC-MA solutions under the dispersion phase flow rate of 0.1 mL/h and continuous phase flow rate of 3 mL/h. B) Images of particles produced from of 5 wt% and 10 wt% HPC-MA droplets. Because of the low concentration of HPC, the assembly process takes extremely long time at room temperature, so the images here show particles produced by heating the droplets at 60℃. (Scale bars: 200 µm)


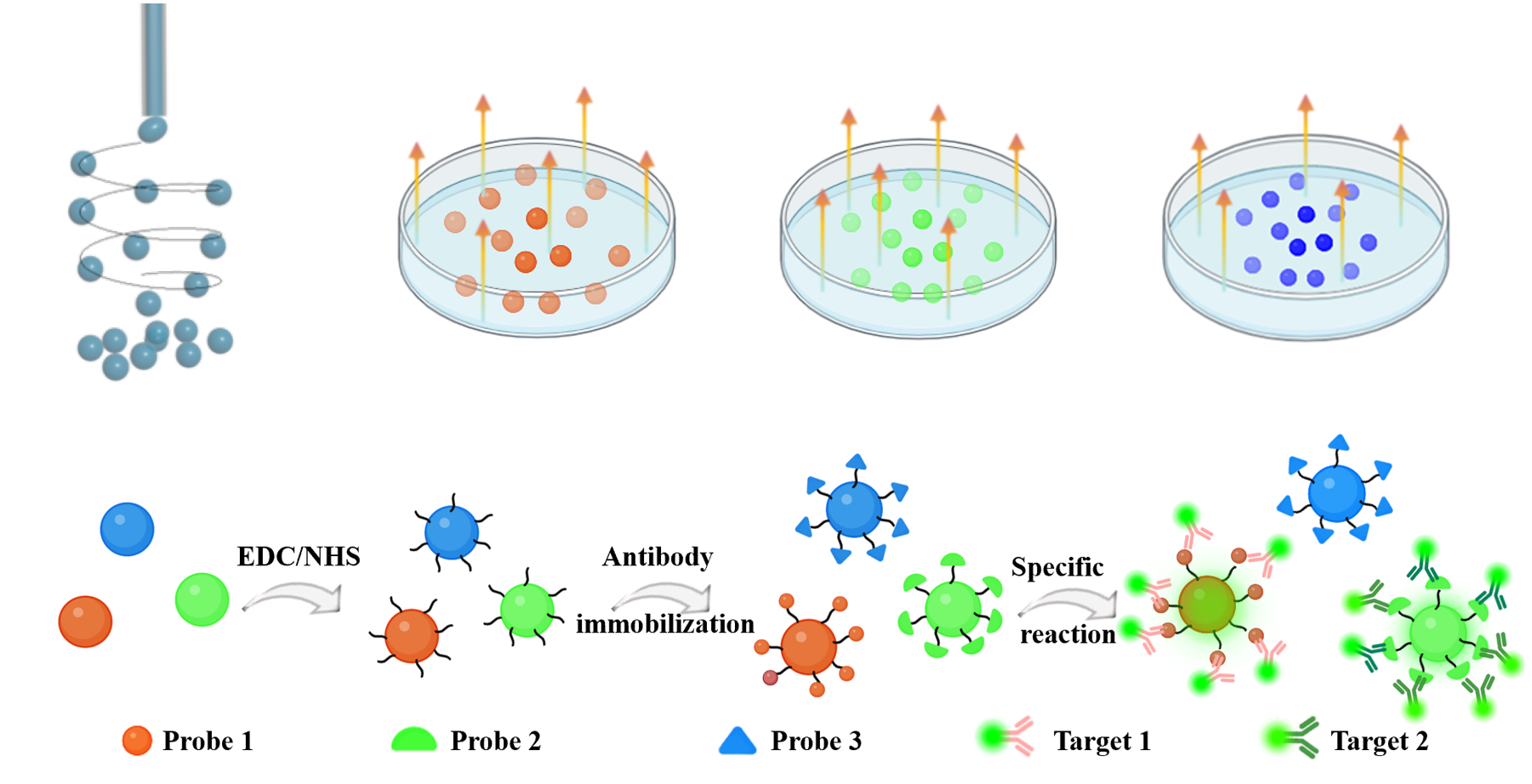


A high-speed revolving microfluidic platform is developed for emulsifying high-viscosity HPC-MA solution to form droplets, which can be self-assembled into CLCs by water evaporation and cured as structure color particles with well-defined and adjustable encoding information while maintaining excellent biocompatibility. The prepared barcode particles demonstrate great potential in multiplex immunoassay applications.
